# Multimodal Locomotion and Cargo Transportation of Magnetically Actuated Quadruped Soft Microrobots

**DOI:** 10.34133/cbsystems.0004

**Published:** 2022-12-30

**Authors:** Chenyang Huang, Zhengyu Lai, Xinyu Wu, Tiantian Xu

**Affiliations:** ^1^ Guangdong Provincial Key Lab of Robotics and Intelligent System, Shenzhen Institute of Advanced Technology, Chinese Academy of Sciences, Shenzhen 518055, China.; ^2^ SIAT Branch, Shenzhen Institute of Artificial Intelligence and Robotics for Society, Shenzhen 518055, China.; ^3^ University of Chinese Academy of Sciences, Beijing 100049, China.

## Abstract

Untethered microrobots have attracted extensive attention due to their potential for biomedical applications and micromanipulation at the small scale. Soft microrobots are of great research importance because of their highly deformable ability to achieve not only multiple locomotion mechanisms but also minimal invasion to the environment. However, the existing microrobots are still limited in their ability to locomote and cross obstacles in unstructured environments compared to conventional legged robots. Nature provides much inspiration for developing miniature robots. Here, we propose a bionic quadruped soft thin-film microrobot with a nonmagnetic soft body and 4 magnetic flexible legs. The quadruped soft microrobot can achieve multiple controllable locomotion modes in the external magnetic field. The experiment demonstrated the robot’s excellent obstacle-crossing ability by walking on the surface with steps and moving in the bottom of a stomach model with gullies. In particular, by controlling the conical angle of the external conical magnetic field, microbeads gripping, transportation, and release of the microrobot were demonstrated. In the future, the quadruped microrobot with excellent obstacle-crossing and gripping capabilities will be relevant for biomedical applications and micromanipulation.

## Introduction

Untethered microrobots have received much attention for their potential in biomedical applications and small-scale micromanipulation [1–6]. Microrobots are usually actuated by external energy source because of their small size and the difficulty of on-board power, such as magnetic fields, ultrasound, electric fields, light, cell-driven devices, and chemical fuels [7–11]. Among the numerous actuation methods, magnetic fields are widely used to actuate microrobots for biomedical applications because of the fact that magnetic fields are harmless to biological cells and tissues. In particular, low-density magnetic fields can be easily generated by electromagnetic coils, and many types of magnetic fields can be achieved by controlling the coil current [12].

Magnetically actuated microrobots for biomedical applications are widely studied [13,14]. Nelson et al. [15] proposed a shuttle-shaped microrobot toward targeted retinal drug delivery, which can be injected into the eye and actuated and steered by an external magnetic field. Zhang et al. [16] proposed a magnetic helical micromachine that can be used to transport cargo in viscous liquids, which contains a head of a cargo holder and a magnetic helical tail. Xu et al. [17–21] designed path-following control methods for magnetic helical swimming microrobots in 2-dimensional and 3-dimensional viscous liquid spaces with high control accuracy. Although this magnetic helical microrobot has the potential to target drug transport in the viscous blood environment, its rigid structure and single mode of motion limit its application.

Compared with rigid structures, microrobots with soft structures exhibit adaptive bionic locomotion in unstructured complex and harsh environments, such as biological digestive tracts, intestines, stomach cavities, bladders, and curved blood vessels [22–31]. Inspired by multimodal locomotion and adaptive functions of octopus, Du et al. [32] reported a soft millirobot with a magnetic head and a functional tail that demonstrated great environmental adaptability for traversing obstacles, deformation, and color change in unstructured environments. Inspired by the locomotion of the scyphomedusae ephyra, Ren et al. [33] proposed an untethered jellyfish-like soft microrobot consisting of 8 magnetically elastic pendant lappets and a nonmagnetic central bubble that can achieve jellyfish-like swimming under an external magnetic field. Inspired by the swimming of zebrafish, Huang et al. [34] demonstrated a magnetically actuated miniature robotic fish with a flexible magnetic skeleton and a soft nonmagnetic body that can swim flexibly in liquid. Hu et al. [35] proposed a magnetically actuated soft millimeter-scale robot with multimodal motion, which can swim inside and on surfaces of liquids, climb liquid meniscus, roll and walk on solid surfaces, jump over obstacles, and crawl through narrow tunnels. Although magnetically actuated soft microrobots inspired by the locomotion of legless creatures have demonstrated excellent multimodal locomotion in unstructured environments, footed magnetically actuated microrobots that walk like quadrupeds still present challenges.

Magnetically actuated soft microrobots have shown excellent capabilities for micromanipulation at the microscale, which implies great potential for targeted drug transport and cell manipulation [36–40]. Floyd et al. [41] present 2 methods of micromanipulation of underwater microspheres using an untethered electromagnetically actuated magnetic microrobot, including the physical direct contact manipulation method and the fluid indirect manipulation method. Su et al. [42] proposed a cruciform thin-film microrobot that enables microbead gripping and transport. Soft microrobots are widely used in micromanipulation tasks because of their excellent deformability and minimal invasion to the target objects.

**Fig. 1.**
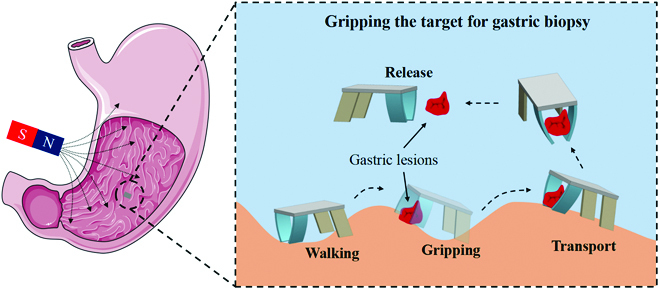
Schematic diagram of the bionic walking and gripping of the magnetically actuated quadruped soft microrobot toward gastric biopsy.

In this work, we propose a bionic quadruped soft thin-film microrobot toward gastric biopsy, which contains a nonmagnetic soft body and 4 magnetic flexible legs (Fig. 1). The quadruped soft microrobot can achieve multiple controllable locomotion modes in an external magnetic field, such as walking like a quadruped animal on the surface and rolling like a wheel. The motion principle and motion model of the microrobot in the actuated magnetic field are presented and verified by deformation characteristic experiments and velocity characteristic experiments. The experiment demonstrates that the microrobot walks through multistep steps 3 mm high and can also move adaptively on the surface of a pleated stomach model. This suggests that the microrobot has the potential to move and perform tasks within the complex stomach environment. The experiments also demonstrated the grasping, transporting, and releasing of microbeads by the microrobot, which facilitates future grasping tasks for performing microrobotic stomach biopsies.

## Materials and Methods

### Design and fabrication of the quadruped microrobot

Inspired by intelligent creatures in nature, we designed and fabricated a new untethered quadruped soft thin-film microrobot consisting of 4 magnetic soft legs with special magnetized profiles and a nonmagnetic film body.

**Fig. 2.**
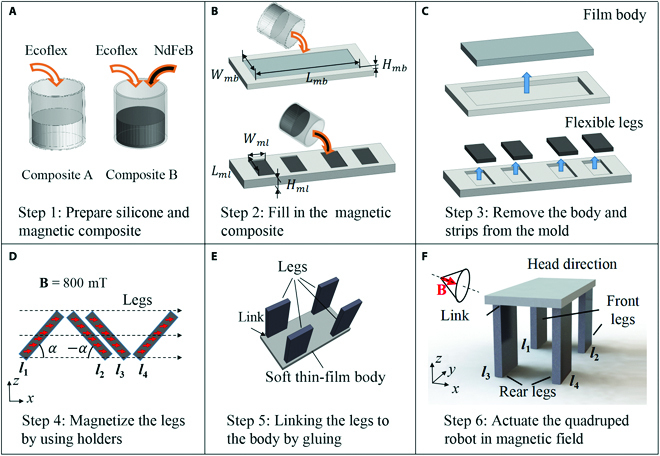
Schematic process of fabrication and magnetization of the quadruped soft microrobot. (A to C) Composite A (pure Ecoflex) and composite B (Ecoflex + NdFeB) were filled into the 3D-printed molds to obtain the robot’s film body and the robot’s 4 flexible legs by the demold method. (D) The 4 legs were magnetized by placing them in the magnetizer (B = 800 mT) at a special inclination angle *α*. (E) Composite A was used as a glue to link the legs to the body. (F) The quadruped soft microrobot moves in the external magnetic field.

The quadruped soft thin-film microrobot was fabricated by demolding technology, and the molds were produced by high-precision 3-dimensional (3D) printing process (Fig. 2). Firstly, we prepared 2 mixed composite liquids, where composite A is made of hard magnetic neodymium-iron-boron (NdFeB) microparticles (average diameter: 46.5 ± 17.6 μm) and the soft silicone rubber (Ecoflex 00-30) with a mass ratio of 1:0.5, and composite B is made from the silicone rubber (Fig. 2A). Then, 2 molds with striped volume used to fabricate the film body and flexible legs were made by 3D printing equipment and coated with a thin layer of resin to make their surface smooth. The first mold with striped volume (length *L_mb_*: 9.5 mm, width *W_mb_*: 4.5 mm, height *H_mb_*: 0.5 mm) is filled with composite A, and the second mold with striped volumes (4 volumes, each with length *L_ml_*: 5.5 mm, width *W_ml_*: 2.5 mm, height *H_ml_*: 0.5 mm) is filled with composite B (Fig. 2B). Each mode is then placed in a warm oven to allow the silicone to fully cure. After curing and cooling to room temperature, the film body and flexible legs can be easily peeled from the mold without being damaged (Fig. 2C). The magnetic particles in the flexible legs are not premagnetized and cannot move in the external magnetic field. To program the magnetization profile of the robot’s flexible legs, we placed the robot’s 4 legs on the supports with specific inclination angle *α* and applied a strong magnetic field with a density of 800 mT (Fig. 2D). The 4 flexible magnetic legs of the robot have a special magnetization profile, with the front leg *l*_1_ and the rear leg *l*_3_ both having a magnetization angle of *α* = 45°, and the front legs *l*_2_ and *l*_4_ both having a magnetization angle of –*α* = 45°. In the same external magnetic field, the deformation response of the 2 front legs or the 2 rear legs is symmetric because the magnetization direction between them is symmetric. Each leg is firmly anchored to the robot body by links and exhibits magnetoelastic bending in response to external magnetic fields (Fig. 2E). Finally, the obtained quadruped soft thin-film microrobot can be driven in an external periodically time-varying magnetic field (Fig. 2F).

### Elastic-magnetic bending model

**Fig. 3.**
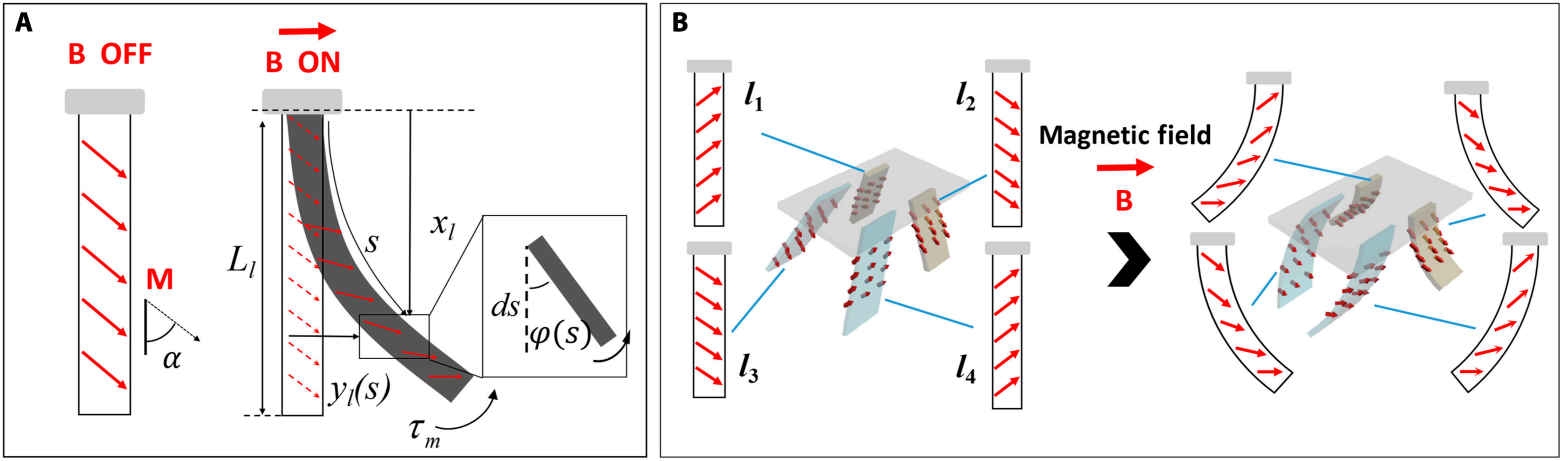
Elastic-magnetic bending model of the magnetic leg of the robot. (A) Deformation analysis of the flexible leg in the magnetic field. (B) Deformation analysis of the quadruped soft microrobot in the magnetic field.

The magnetic flexible legs of the quadruped soft microrobot with magnetic properties are subjected to magnetic forces and torques from the external actuating magnetic field. In the uniform magnetic field, all magnetic objects will be exposed to a negligible magnetic force and a magnetic torque with corresponding magnitude, which can be expressed as

$$\tau_{m}=V_{m}M\times B$$ (1)

where *V_m_*, **M** and **B** are the volume of the magnetic object, the magnetization of the magnetic object, and the flux density of the uniform magnetic field. Therefore, the deformation of each magnetic leg of the robot in the external magnetic field can be expressed by an elastic-magnetic bending model. To simplify the model, the leg frame $L_{F}=\left\{x_{l}\;y_{l}\;z_{l}\right\}$ is defined in Fig. 3A, where *x_l_*, *y_l_*, and *z_l_* are along the direction of the length, width, and thickness of the leg, respectively. The magnetization profile of the magnetic leg of the robot can be expressed as

$$M=M{\left[\cos\;\alpha \;\sin\;\alpha \;0\right]}^{T}$$ (2)

where *M* and *α* are the magnetization magnitude and the magnetization angle, respectively. The magnetic flexible leg can be considered as a cantilever beam with 1 end fixed and 1 end free and deformed by the external magnetic torque. When the deflection is small, the bending moment of the magnetic flexible leg can be expressed by the Euler-Bernoulli equation as

$$\tau_{m}A\left(s\right)=\mathrm{EI}\frac{\partial^{2}\varphi }{\partial s^{2}}$$ (3)

where *A* is the cross-sectional area, *I* is the moment of inertia and *s* is the distant along the long axis of the leg. *φ* = dy*_l_*/*dx_l_* is the rotational deflection along the leg. We assume that the magnetic field $B={\left[0\;B\;0\right]}^{T}$ is parallel to the *y_l_*−axis at a certain moment. Then, the boundary conditions include *y_l_*(0) = 0 and *y_l_*(*L_l_*) = *π*/2 − *α*. Therefore, according to Eqs. 1 to 3, the vibration equation of the flexible leg can be expressed as

$$y_{l}\left(x\right)=\frac{\mathrm{mA}L_{l}^{3}B}{\kappa^{3}\mathrm{EI}}\sin\;\left(\frac{\kappa }{L_{l}}x\right)$$ (4)

where *κ* = *π*/2 − *α* is the residual angle of *α*. It can be seen that the magnitude of the deformation is positive correlation to the magnitude of the magnetic field *B*.

### Locomotion of the quadruped microrobot

**Fig. 4.**
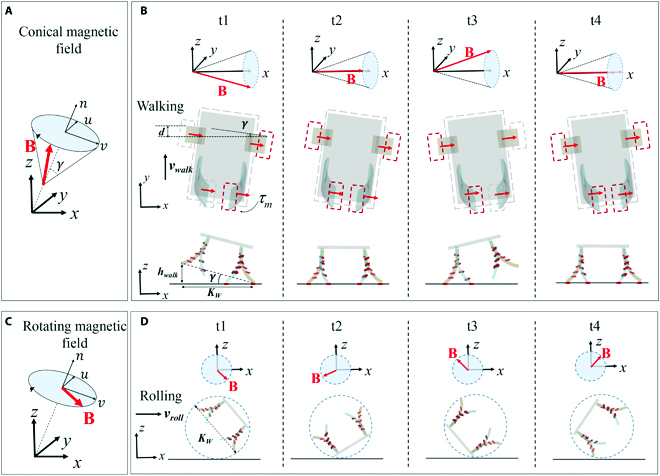
Locomotion modes of the quadruped soft microrobot in magnetic field. (A and B) When a conical magnetic field is applied, the robot can walk on surface. (C and D) When a rotating magnetic field is applied, the robot can roll on surface.

On the basis of the principle of deformation of the robot’s magnetic leg in the external magnetic field, the stable gait of locomotion of the microrobot can be achieved by programming the actuating magnetic field. By using different actuating magnetic fields, the quadruped microrobot can achieve multiple locomotion modes, such as walking and rolling, as shown in Fig. 4.

By generating a conical magnetic field, the microrobot can alternately move its legs to achieve walking on a flat surface, just like the gait of a quadruped (Fig. 4A and B). The conical magnetic field is defined as the superposition of the rotating magnetic field and constant magnetic field, which can be expressed as Fig. 4A

$$B\left(t\right)=B{\left[\sin\;\left(\gamma \right)\cos\;\left(2\mathrm{\pi ft}\right)u+\sin\;\left(\gamma \right)\sin\;\left(2\mathrm{\pi ft}\right)v+\cos\;\left(\gamma \right)n\right]}^{T}$$ (5)

where the angle *γ* is defined as the angle between the magnetic field **B** and the direction of the conical centerline. *f* is the frequency of the conical magnetic field. The unit vector *n* is the direction vector that represents the conical centerline in the world coordinate frame. The unit vectors *u* and *ν* represent the base vectors of the rotating bottom plane in the world coordinate frame. The gait of the quadruped microrobot can be simplified as shown in Fig. 4B. The step length *d* is related to the angle *γ*, which can be expressed as

$$d=K_{W}\sin\;\gamma$$ (6)

where *K_W_* is the distance between the 2 front legs of the robot. *K_W_* can be obtained by adding the magnetic deformation of the legs (according to Eq. 4) to the initial distance between the 2 legs, which can be expressed as

$$\begin{array}{cc} K_{W} & =W_{b}+2y_{l}\left(L_{l}\right)\\ & =W_{b}+\frac{2\mathrm{mA}L_{l}^{3}B}{\kappa^{3}\mathrm{EI}}\sin\;\left(\kappa \right) \end{array}$$ (7)

At 1 cycle of the conical magnetic field, both the left and right legs move the robot forward by *d*, and then the walking velocity *υ_walk_* can be approximated as

$$v_{\text{walk}}=2d=2K_{W}f_{w}\sin\;\gamma$$ (8)

where *f_w_* is the frequency of the walking motion, which is usually equal to the rotating frequency of the external conical magnetic field which is below the step-out frequency. Moreover, the walking robot can straddle high obstacles, because the robot’s legs can be lifted to a height *h_walk_*, which can be expressed as

$$h_{\text{walk}}=K_{W}\sin\;\gamma$$ (9)

By generating a rotating magnetic field, the microrobot realizes rolling on a flat surface. (Fig. 4C and D). The rotating magnetic field **B** is defined as the magnetic field rotates around a unit vector $n={\left[n_{x}\;n_{y}\;n_{y}\right]}^{T}$ in the 3D space. The rotating **B** can be expressed as

$$B\left(t\right)=B{\left[\cos\;\left(2\mathrm{\pi ft}\right)u+\sin\;\left(2\mathrm{\pi ft}\right)v\right]}^{T}$$ (10)

where **u** and **v** represent the base vectors in the rotating plane of **B**, which are all perpendicular to **n**. The gait of the quadruped microrobot can be simplified as shown in Fig. 4D. At 1 cycle of the rotating magnetic field, the step length of the robot’s rolling motion is the distance *K_W_* of the 2 front legs when the frequency of the rotating magnetic field is below the step-out frequency. Therefore, the rolling velocity *υ_roll_* can be approximated as

$$v_{\text{roll}}=K_{W}f$$ (11)

where *f* is the frequency of the rotating magnetic field.

### Gripping of the quadruped microrobot

**Fig. 5.**
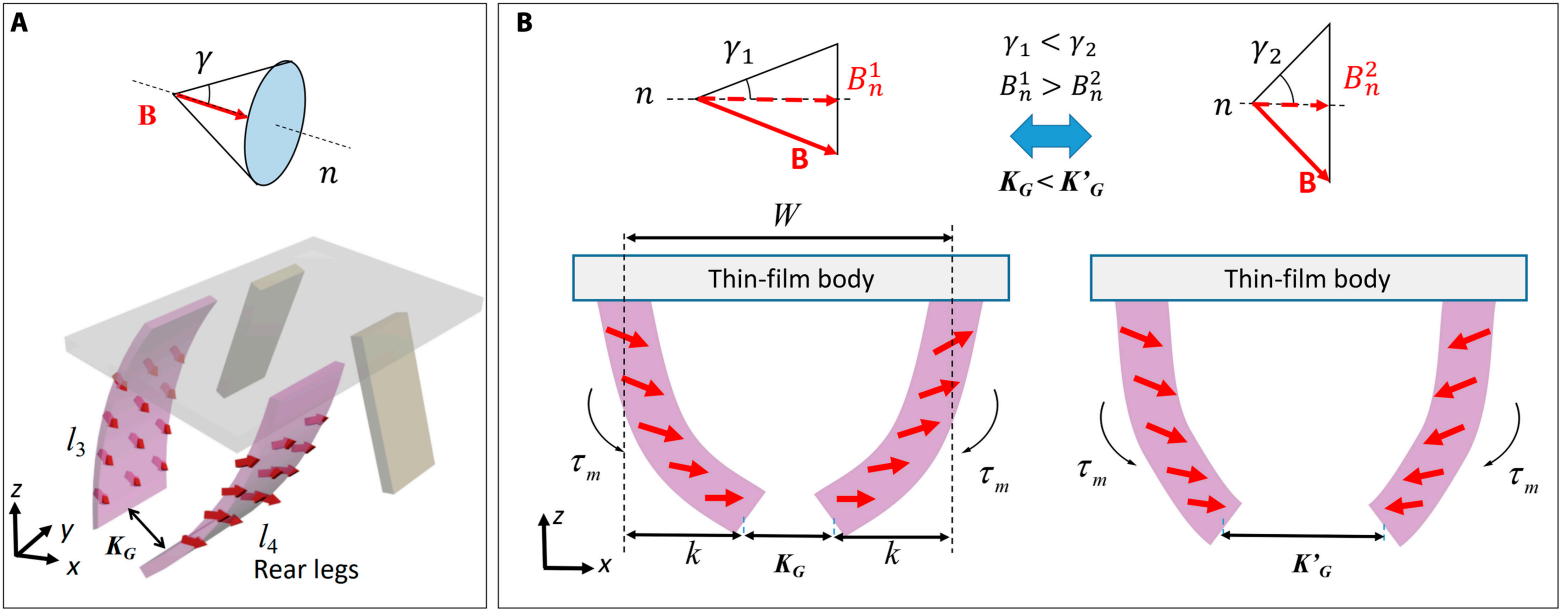
Gripping behavior of the quadruped soft microrobot. (A) Driven by the conical magnetic field, the microrobot’s rear legs can act as grippers to grip cargo. (B) By controlling the conical angle *γ* of the external conical magnetic field, the distance of the robot’s rear legs can be adjusted.

The robot’s front legs have a greater distance, allowing for faster movement speeds and better obstacle-crossing capabilities. The distance between the rear legs of the robot can be adjusted by controlling the component of the external magnetic field in the lateral direction of the robot body to further realize the grasping, transporting, and releasing of cargo. For the quadruped microrobot, cargo gripping can be achieved when a conical magnetic field with a small conical angle *γ* is applied. When keeping the conical angle *γ* of the magnetic field constant, cargo transport can be achieved. When a conical magnetic field with a larger *γ* is applied, cargo release can be achieved. The gripping behavior of the robot’s rear legs is shown in Fig. 5, and the distance *K_G_* of the rear legs can be expressed as

$$\begin{array}{cc} K_{G} & =W-2k=W-\frac{2\mathrm{mA}L_{l}^{3}B_{n}}{\kappa^{3}\mathrm{EI}}\sin\left(\kappa \right)\\ & =W-\frac{2\mathrm{mA}L_{l}^{3}B}{\kappa^{3}\mathrm{EI}}\sin\left(\kappa \right)\cos\left(\gamma \right) \end{array}$$ (12)

where *W* is the initial distance between the 2 rear legs, which can be approximated as the film body width *W_b_*. *k* is the deformation distance of the end of the rear leg, which can be found by Eq. 4. *B_n_* is the component of the magnetic field **B** in the direction of the central n-axis.

## Results and Discussion

### Experimental setup

**Fig. 6.**
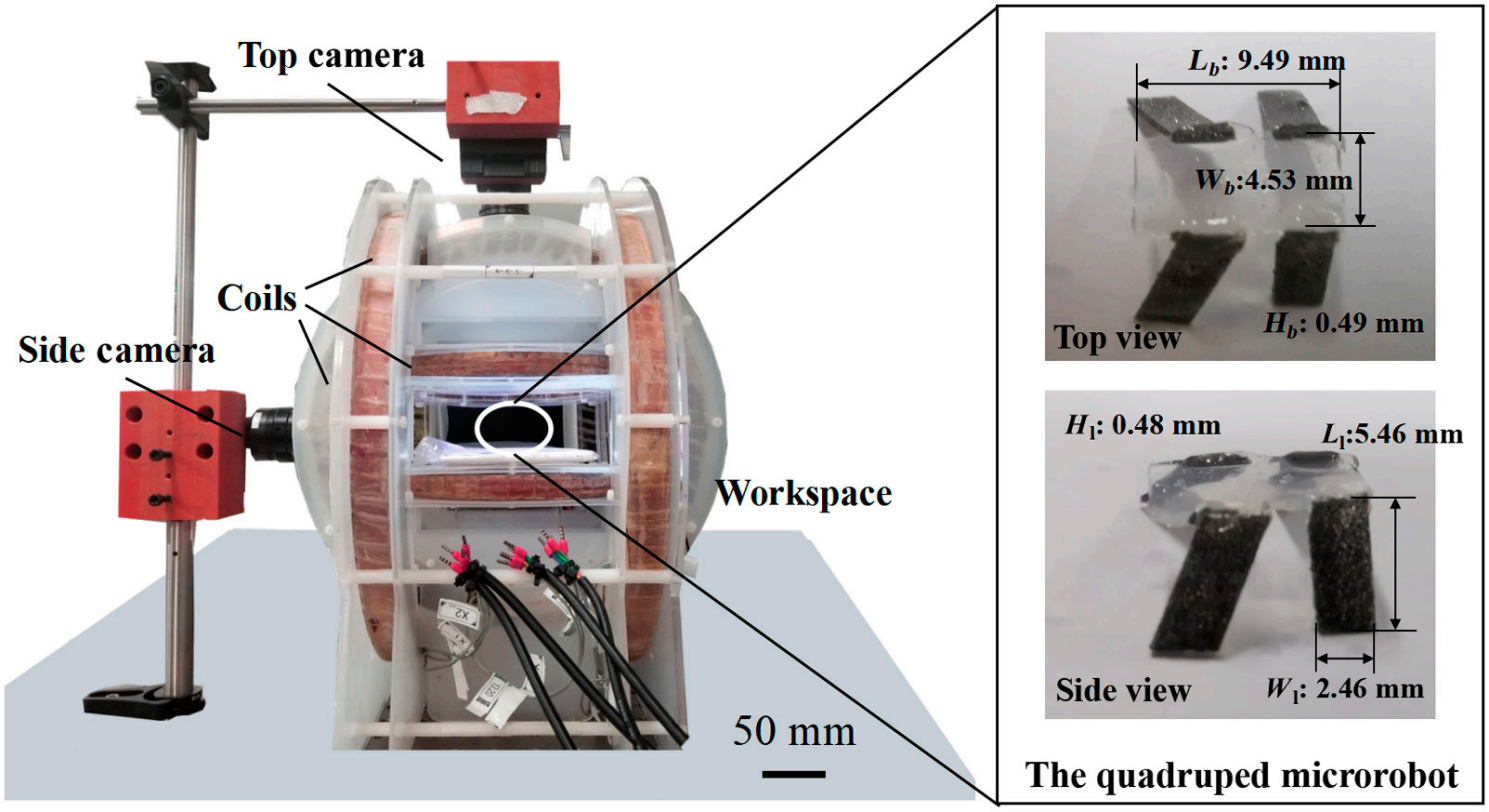
The electromagnetic coils system and the quadruped soft microrobot.

The electromagnetic actuation system was developed and assembled to actuate and control the microrobot, which contains an electromagnetic coil module, a visual positioning module, and a human–machine interaction module (Fig. 6). The electromagnetic coil module is used to generate an arbitrary periodic magnetic field, which contains 3 pairs of Helmholtz coils distributed orthogonally on the central axis. The magnetic field density generated by each pair of coils is approximately linearly mapped to the current magnitude. The current in each pair of coils can be controlled by the computer with a digital-to-analog input/output converter (Model 826, Sensoray, USA), a servo controller (ESCON 70/10 motor drivers, maxon, Switzerland), and a dc power supply (IT6000B, ITECH, China). The vision positioning module is used to measure the position and pose of the miniature robot in real time, which contains 2 high-speed cameras (Blackfly S BFS-U3-16S2M, FLIR Systems, USA) placed on the top and side of the system. The human–machine interaction module is used to deliver control commands from the user to the robot and includes a graphical user interface (graphical user interface programming with Qt5 and C++) and an interaction joystick (Xbox Wireless Controller, Microsoft, USA). The microrobot can move in a viscous silicone fluid, which has a viscosity of 20 cst if not otherwise specified. Top and side views of the quadruped microrobot in a liquid sink in the workspace are captured by the 2 cameras, as shown in Fig. 6.

### Deformation characteristics of the magnetic flexible legs

**Fig. 7.**
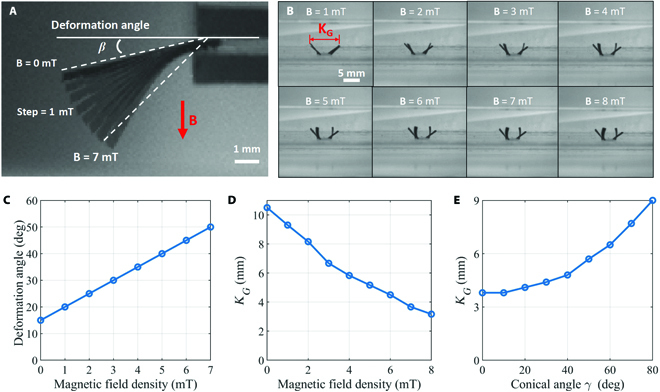
Deformation analysis of the flexible magnetic leg of the quadruped soft microrobot. (A and C) Relationship between the deformation angle of the leg and the density of the external magnetic field. (B to D) Relationship between the legs’ distance and the density and the conical angle of the magnetic field.

The deformation of the magnetic legs of the robot was measured when different magnitudes of magnetic fields were applied, as shown in Fig. 7A. Firstly, 1 end of the robot’s leg is held by the clamp and the other end is free. The angle *β* between the line connecting the 2 ends of the robot leg and the horizontal plane is defined as the deformation angle. Because of gravity, the flexible soft microrobot leg will be free to droop at an angle of 18° when no magnetic field is applied. Then, a magnetic field **B** in the vertical direction from 0 to 7 mT was applied in steps of 1 mT, and the deformation angle of the robot leg was measured. The relationship between the leg deformation angle and the magnitude of the magnetic field is shown in Fig. 7C. The experimental results show that the deformation angle of the robot leg increases with the increase of the magnitude of the magnetic field, which is also expected from our theoretical model. When the external magnetic field **B** is 7 mT, the deformation angle of the robot increases from the initial 18° to 46°.

### Velocity characteristics and steering of the quadruped microrobot

**Fig. 8.**
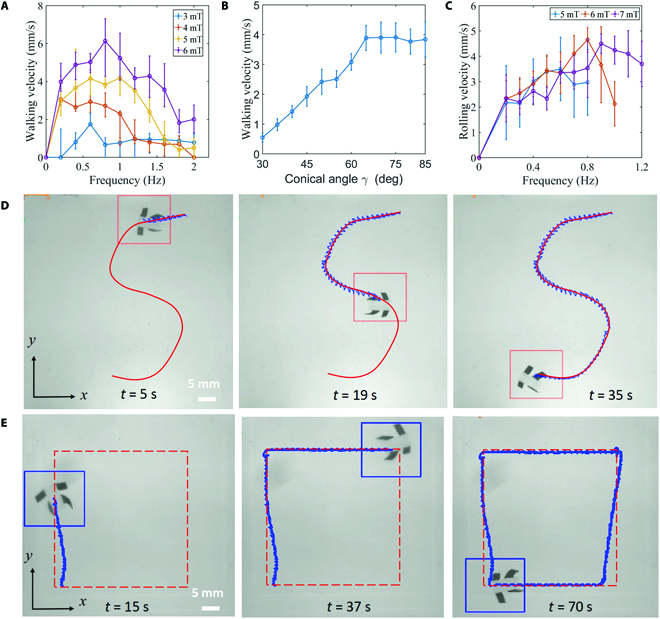
Velocity characteristics and steering of microrobot. (A) Walking velocity of the robot as a function of the frequency of the conical field. (B) Walking velocity of the robot as a function of the conical angle *γ* of the conical field. (C) Rolling velocity of the robot as a function of the frequency of the rotating field. (D and E) Steering control of the quadruped soft microrobot.

The walking velocity of the microrobot is related to the external conical magnetic field parameters, as shown in Fig. 8A and B. The conical angle *γ* of the external conical magnetic field is kept at 65°, its frequency varies from 0 to 2 Hz in steps of 0.2 Hz, and its magnetic field density varies from 3 to 6 mT in steps of 1 mT, as shown in Fig. 8A. The experimental results show that the quadruped microrobot can walk at a greater velocity when the conical magnetic field with a greater magnetic field density is applied, which is because a larger **B** allows the robot to have longer step lengths according to Eqs. 4 and 6 at 1 motion cycle. In addition, the walking velocity of the robot increases with the frequency of the external conical magnetic field **B**, and the speed of the robot decreases when the frequency of **B** exceeds the step-out frequency. As shown in Fig. 8B, when a conical magnetic field with a larger conical angle *γ* is applied, the microrobot has a larger walking velocity and has a maximum speed of 3.90 mm/s in the field **B** with the conical angle *γ* of 65°. When the conical angle *γ* of **B** increases to greater than 65°, the walking speed of the microrobot remains approximately constant. According to Eq. 8, the application of a conical magnetic field with a larger conical angle will result in a higher walking speed, but the rotational angular velocity of the robot will not be synchronized with the angular velocity of the magnetic field because of the viscous drag.

The rolling velocity of the microrobot is related to the external rotating magnetic field parameters, as shown in Fig. 8C. The frequency of the rotating magnetic field varies from 0 to 1.2 Hz in steps of 0.2 Hz, and its magnetic field density varies from 5 to 7 mT in steps of 1 mT. Because of the viscous resistance received in viscous liquids, rotating magnetic fields with particularly small densities and particularly high frequencies will not be able to drive the microrobot to achieve rolling motion. As shown in Fig. 8C, the rolling speed of the robot increases with the increase of the magnetic field frequency when the frequency is below the step-out frequency. The experimental results show that the rolling motion of the microrobot in the rotating magnetic field with greater density has a step-out frequency.

The quadruped microrobot can walk along a predetermined reference path with S shapes and square shapes controlled by steering controller, as shown in Fig. 8D and E. By regulating the direction of the n-axis of the external conical magnetic field, flexible steering control of the microrobot can be achieved. In the future, closed-loop control methods with visual feedback will be applied to paths following control of the quadruped microrobot.

### Locomotion in complex environments

**Fig. 9.**
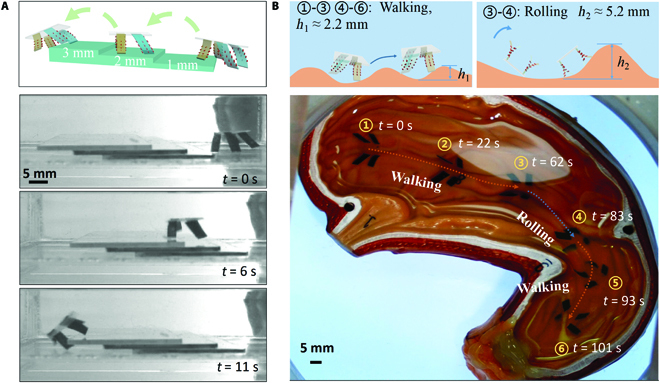
(A) The quadruped microrobot walks across 3 flights of steps. (B) The quadruped microrobot moves on the bottom of the stomach model filled with grooves.

The quadruped microrobot not only has stable multiple movement modes and flexible steering control but also can move in a variety of complex obstacle environments. As shown in Fig. 9A, the quadruped microrobot can walk across 3 levels of steps, each with a height of 1 mm. In particular, as seen in Movie S1, the robot has a steady speed over the obstacles while keeping its body from falling. The quadruped microrobot can also be used in the future to perform tasks in complex environments such as natural cavities. As shown in Fig. 9B, the quadruped microrobot can move on the bottom of the stomach model filled with grooves. The microrobot can walk over some low obstacles that are approximately 2.2 mm high. When the height of the obstacle is large, the microrobot can switch to rolling mode to cross the obstacle.

### Gripping and transporting cargo

**Fig. 10.**
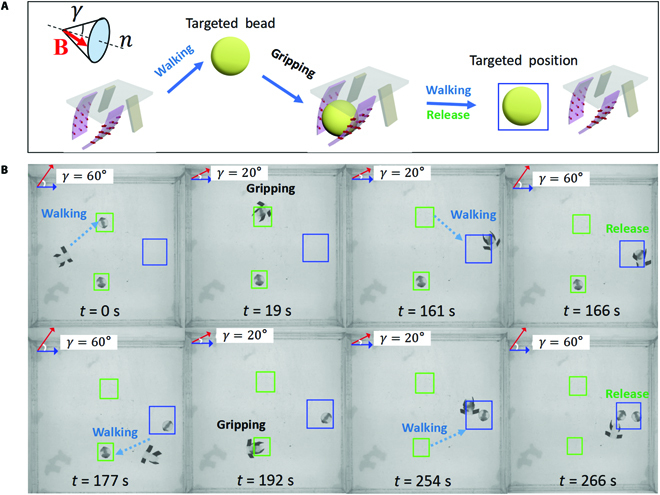
(A) Schematic diagram of the quadruped microrobot gripping, transporting, and releasing cargo. (B) The quadruped microrobot grips 2 targeted beads in different locations in turn and transports them to the targeted position for release.

The quadruped microrobot not only has excellent obstacle-crossing capability but also has excellent cargo gripping capability. The magnitude of the magnetic field component in the lateral direction of the microrobot body enables the adjustment of the distance between the rear legs of the microrobot, which enables the gripping and releasing of cargo, as shown in Fig. 10A. The ability to grip and transport the cargo was experimentally verified by sequentially transporting 2 target beads in different positions to the specified target location, as shown in Fig. 10B. Each microbead has a diameter of 6 mm and a mass of 127.3 mg, while the microrobot itself weighs 41.1 mg. During the whole experiment, the density of the external conical magnetic field was set to 5 mT and the frequency was set to 1.2 Hz. Firstly, the microrobot walks to the vicinity of the first target bead in the conical magnetic field with a conical angle amma of 60°. When the robot body wraps the bead, the *γ* is set to 20° thereby enabling the gripping of the bead. The robot walks to the target position in such conical magnetic field with *γ* of 20°. When the robot reaches the target position, the γ of the magnetic field is set to 60°, thus releasing the bead. We repeated the above process to achieve the gripping, transporting, and releasing of the second bead. Experimental results show that the microrobot can grasp and transport objects up to 3 times its own weight.

## Conclusion

In this work, we have proposed a bionic quadruped soft thin-film microrobot with a nonmagnetic soft body and 4 magnetic flexible legs. We first introduced the process of making the robot and the magnetization process. The deformation of the magnetic leg of the robot in the external magnetic field has been modeled and analyzed. The mechanism of multiple locomotion modes of the quadruped microrobot in the external magnetic field has been analyzed and modeled. In addition, we have presented the mechanism by which the microrobot grips the cargo and transports it by its hind legs. In the experiment, the deformation characteristics of the robot’s legs and the velocity characteristics of the robot’s motion were analyzed. The controlled steering capability of the robot was experimentally verified by following given reference paths. The experiments demonstrated the excellent obstacle-crossing ability of the microrobot, such as traversing steps with a height of 3 mm and a stomach model with multiple gullies. Finally, the experiment demonstrates the microrobot transporting multiple microbeads from different locations to the target position. In future work, we will optimize the microrobot design toward more efficient motion and better gripping capabilities. In addition, autonomous motion control and gripping control of quadruped microrobots are to be investigated in a biopsy task.

## Data Availability

All data needed to evaluate the conclusions in the paper are present in the paper and/or the Supplementary Materials. Additional data related to this paper may be requested from the authors.

## Supplementary Materials

Supplementary Materials Movie S1. (1) Two locomotion modes of the quadruped soft microrobot. (2) Steering the robot to follow reference paths. (3) Locomotion in complex obstacle environments. (4) Transportation of microbeads.
